# Phenotypic characterization of aberrant stem and progenitor cell populations in myelodysplastic syndromes

**DOI:** 10.1371/journal.pone.0197823

**Published:** 2018-05-25

**Authors:** Benjamin N. Ostendorf, Eva Flenner, Anne Flörcken, Jörg Westermann

**Affiliations:** 1 Department of Hematology, Oncology, and Tumor Immunology, Charité –Universitätsmedizin Berlin, Campus Virchow Klinikum, Berlin, Germany; 2 Labor Berlin Charité Vivantes GmbH, Berlin, Germany; University of Campinas, BRAZIL

## Abstract

Recent reports have revealed myelodysplastic syndromes (MDS) to arise from cancer stem cells phenotypically similar to physiological hematopoietic stem cells. Myelodysplastic hematopoiesis maintains a hierarchical organization, but the proportion of several hematopoietic compartments is skewed and multiple surface markers are aberrantly expressed. These aberrant antigen expression patterns hold diagnostic and therapeutic promise. However, eradication of MDS requires targeting of early myelodysplasia propagating stem cells. This warrants an exact assessment of the differentiation stage at which aberrant expression occurs in transformed hematopoiesis. Here, we report results on the prospective and extensive dissection of the hematopoietic hierarchy in 20 patients with either low-risk MDS or MDS with excess blasts and compare it to hematopoiesis in patients with non-malignancy-associated cytopenia or B cell lymphoma without bone marrow infiltration. We found patients with MDS with excess blasts to exhibit characteristic expansions of specific immature progenitor compartments. We also identified the aberrant expression of several markers including ALDH, CLL-1, CD44, and CD47 to be specific features of hematopoiesis in MDS with excess blasts. We show that amongst these, aberrant CLL-1 expression manifested at the early uncommitted hematopoietic stem cell level, suggesting a potential role as a therapeutic target.

## Introduction

Myelodysplastic syndromes (MDS) comprise a heterogeneous group of clonal hematopoietic disorders leading to ineffective hematopoiesis. Recent reports have revealed the existence of MDS propagating stem cells [1–5]. While the hematopoietic hierarchy is conserved in MDS, hematopoietic stem and progenitor cell (HSPC) populations were shown to be skewed [2,4,5].

Cancer stem cells are suggested not only to initiate malignancies but also to constitute a pool of quiescent cells that can hardly be eliminated by conventional therapy [3]. Thus, the elimination of cancer stem cells is deemed to be both essential and sufficient to eradicate cancer. The identification of aberrant surface markers on cancer cells has fueled considerable efforts to develop specific antineoplastic therapies. However, in order to target cancer propagating stem cells, a precise assessment of the timing of aberrant expression of individual markers during malignant hematopoiesis is required. Many aberrant cell surface markers have been identified in MDS (reviewed in [6]), including recent work reporting the expression of IL-1 receptor accessory protein (IL1RAP) and CD99 on myelodysplastic stem cells [7,8]. In the case of other putative targets, the expression has not been tracked to the exact differentiation stage within the progenitor cell compartment [9]. In addition, several markers known to be aberrantly expressed in other myeloid malignancies such as acute myeloid leukemia (AML), have so far not been assessed in MDS [10,11].

Here, we present the prospective and extensive examination of the hematopoietic hierarchy in 20 patients with MDS in comparison with patients exhibiting either non malignancy-associated cytopenia or B-Non-Hodgkin-Lymphoma (B-NHL) without bone marrow infiltration. Building upon recently characterized approaches to delineate all major immature hematopoietic compartments, we identified specific alterations in the composition of HSPC compartments in MDS with excess blasts and showed the aberrant expression of several markers including aldehyde dehydrogenase (ALDH), C-type lectin-like molecule-1 (CLL-1, also known as CLEC12A), CD13/CD33, CD44 and CD47 in a differentiation-stage specific manner. This establishes CLL-1 as a potential therapeutic and ALDH as a a potential diagnostic target in MDS with excess blasts.

## Patients and methods

### Patient samples

Bone marrow samples from 69 patients were collected after informed consent, in accordance with the Declaration of Helsinki and after approval by Charité’s ethics committee. The study did not involve the use of donated tissue from any vulnerable populations. Twenty-one patients were excluded from analysis due to lack of a definitive diagnosis (n = 6) or due to infiltration of the bone marrow with a non-MDS malignancy (n = 15). A diagnosis of MDS was made according to the WHO 2016 classification and based on morphology (cytomorphology of bone marrow aspirate and bone marrow biopsy), flow cytometry, and cytogenetics. The group of patients with MDS included 12 cases without excess blasts (< 5%; hereinafter referred to as “low-risk MDS”) as well as 8 cases of MDS with excess blasts (one case of MDS-EB-1 with 5–9% blasts and seven cases of MDS-EB-2 with 10–19% blasts, respectively; hereinafter also referred to as “high-risk MDS”). Controls consisted of patients with non-MDS related cytopenia (n = 17) and another group with newly diagnosed B-NHL without prior treatment (n = 11). In both of these control groups, morphologic assessment (bone marrow biopsy and bone marrow aspirate) and flow cytometry were performed to rule out a malignant cause in patients with non-MDS related cytopenia, and to exclude bone marrow infiltration in patients with newly diagnosed B-NHL. There were no significant differences in the proportions of hematopoietic compartments between these two control groups (data not shown). For comparisons with MDS patients, all control cases were assessed together.

A total of 48 patients with a median age of 68 years (range: 22–100 years) were included in the final analysis (ratio male:female: 0.65:0.35). There were no significant differences regarding age and gender distribution between MDS groups (median age 70 years, range 33–100; ratio male:female 0.6:0.4) and controls (median age 64 years, range 22–88; ratio male: female 0.68:0.32).

### Flow cytometry

**Fig 1:**
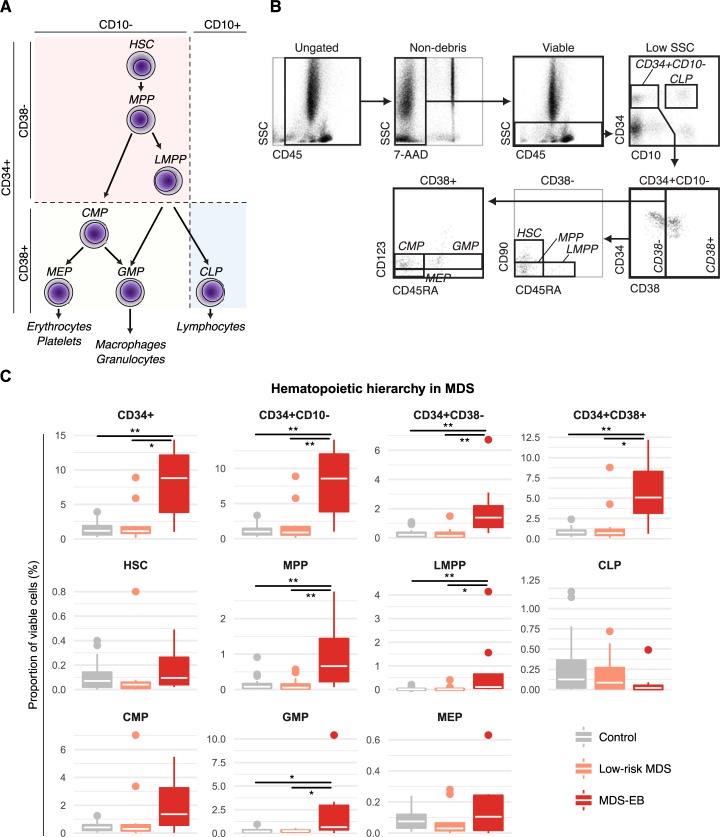
Dissection of all major hematopoietic stem and progenitor cell (HSPC) compartments in MDS reveals expansion of MPP, LMPP, CMP, and GMP compartments in MDS with excess blasts (MDS-EB). **(a)** Schematic representation of major HSPC populations identified by the employed gating strategy. **(b)** Representative example of gating hierarchy. **(c)** Proportions of HSPC compartments in control versus MDS patients expressed as percent of viable bone marrow cells. White crossbars represent the median and boxplots hinges represent the first and third quartiles. *p<0.05, **p<0.01; CLP, common lymphoid progenitor cells; CMP, common myeloid progenitor cells; GMP, granulocyte macrophage progenitor cells; HSC, uncommitted hematopoietic stem cell; LMPP, lymphoid-primed multipotent progenitor cells; MEP, megakaryocytic erythroid progenitor cells; MPP, multipotent progenitor cells.

Samples for flow cytometric analysis were processed within 48 hours after collection. Red blood cells were lysed using commercial lysing buffer (Pharm Lyse, BD Biosciences) according to the manufacturer’s instructions. Antibody stainings were performed for 20 minutes on ice. Flow cytometric identification of uncommitted hematopoietic stem cells (HSC; SSC(lo)CD34+CD10-CD38-CD90+), multipotent progenitor cells (MPP; SSC(lo)CD34+CD10-CD38-CD90-CD45RA-), lymphoid-primed multipotent progenitor cells (LMPP; SSC(lo)CD34+CD10-CD38-CD90-CD45RA+), common lymphoid progenitor cells (CLP; SSC(lo)CD34+CD10+), common myeloid progenitor cells (CMP; SSC(lo)CD34+CD10-CD38+CD123(int)CD45RA-), granulocyte macrophage progenitor cells (GMP; SSC(lo),CD34+CD10-CD38+CD123(int)CD45RA+), and megakaryocyte erythroid progenitor cells (MEP; SSC(lo)CD34+CD10-CD38+CD123-CD45RA-) was performed according to established protocols and as illustrated in Fig 1A and 1B [12–15]. Importantly, as a modification to published approaches we did not perform exclusion of lineage-positive cells owing to a high probability of aberrant expression of lineage markers on myelodysplastic progenitor cells [16–19]. Dead cells were excluded by 7-AAD staining (Beckman Coulter) and isotype controls were used where appropriate. For the analysis of CD44/CD47 expression the geometric mean (mean fluorescence intensity, MFI) was used and reported as log10, since aberrant expression of these markers occurs in a gradual rather than an “on-off” fashion [20,21]. On average, 2237 CD34+ cells were acquired per sample (range, 155–14703).

Staining panels included antibodies for CD45, CD34, CD10, CD38, CD45RA, and either CD90 or CD123 to characterize the hematopoietic hierarchy as outlined above as well as one antibody per test tube for the detection of aberrant antigen expression (CD44, CD47, CLL-1, or combined CD13/CD33) (Table 1). Staining panels for the detection of ALDH included antibodies for CD45, CD34, CD10, and CD38. Antibodies were obtained from Beckman Coulter (anti-CD13 FITC-conjugated, clone SJ1D1; CD123-PC7, clone SSDCLY107D2; CD45RA-PE, clone ALB11; CD38-PB, clone LS198-4-3; CD33-FITC, clone D3HL60.251; CD34-APC, clone 581; CD10-ECD, clone ALB1; CD44-FITC, clone J.173), and BD Biosciences (CD90-PC7, clone 5E10; CD47-FITC, clone B6H12; CLL-1-FITC, clone 50C1). Isotype controls were from Beckman Coulter. For evaluation of ALDH expression Aldefluor staining (Stemcell Technologies) was performed according to the manufacturer's instructions.

**Table 1:** Antibody panels used for flow cytometry analyses.

Panel	PE	ECD	7-AAD	APC	PB	KrOr	PC7	FITC
A	CD45RA	CD10	7-AAD	CD34	CD38	CD45	CD90	One of the following: CD13/CD33, CD47, CD44, CLL-1
B	CD45RA	CD10	7-AAD	CD34	CD38	CD45	CD123	One of the following: CD13/CD33, CD47, CD44, CLL-1
C	CD45RA	CD10	7-AAD	CD34	CD38	CD45	-	ALDH (Aldefluor)

Cells were analyzed using a Navios flow cytometer (Beckman Coulter). Kaluza software (Beckman Coulter) was employed to analyze flow cytometry data. Diagnoses of patients were blinded during gating.

### Statistical analysis

To test for significant differences of continuous variables between multiple groups the non-parametric Kruskal-Wallis rank sum test was employed. Post-hoc testing to compare individual groups was performed using pairwise Wilcoxon rank-sum tests and p-values were false discovery rate-adjusted. To test for significant differences in age between controls and patients with MDS a two-tailed student’s t-test was employed. The Chi-squared test was employed to test for differences in gender distribution between groups. All analyses were performed using R statistical software (The R Foundation for Statistical Computing). The level of significance was 0.05.

## Results

### Quantification of stem and progenitor cell subpopulations in MDS

We first set out to assess the hematopoietic hierarchy in MDS using a modified gating approach without exclusion of lineage-positive cells as described in the methods section (Fig 1A–1B). As expected, patients with MDS-EB showed a significantly enlarged proportion of CD34+ cells, driven by non-lymphoid committed CD10- cells (Fig 1C). This expansion of CD34+CD10- cells was due to an increase of both CD38- and CD38+ populations.

Interestingly, the expansion of the more immature CD34+CD10-CD38- compartment was driven by an increase of both the CD90-CD45RA- MPP and the CD90-CD45RA+ LMPP compartments rather than of uncommitted CD90+CD45RA- HSCs. In contrast, the assessment of more mature CD34+CD10-CD38+ cells revealed an expansion of GMP in patients with MDS-EB. We also assessed the abundance of these progenitor populations in relation to their CD34+CD10-CD38+ parent population. Using this metric, we found the relative proportion of MEPs (proportion MEPs/CD34+CD10-CD38+ cells) to be significantly reduced in both patients with low-risk MDS and MDS-EB as compared to control patients (S1 Fig).

### CLL-1 is upregulated on immature stem cells in MDS with excess blasts

**Fig 2:**
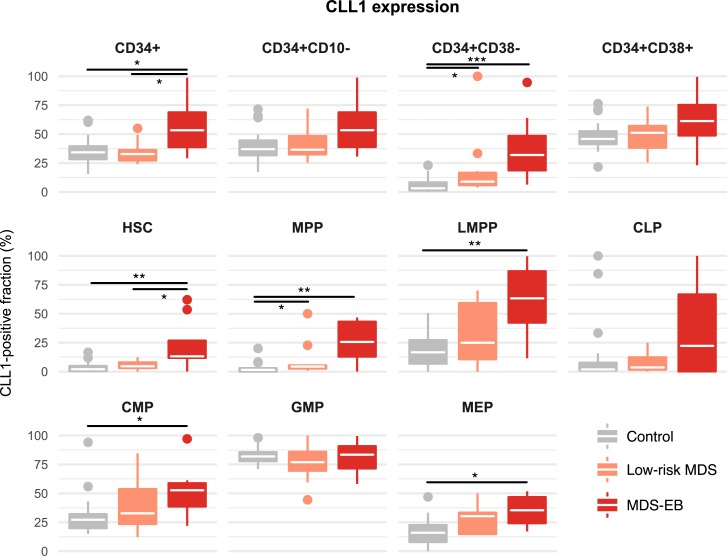
Aberrant CLL-1-expression in MDS with excess blasts manifests at the uncommitted hematopoietic stem cell (HSC) stage and affects multiple progenitor cell compartments. White crossbars represent the median expression and boxplots hinges represent the first and third quartiles. *p<0.05, **p<0.01, ***p<0.001; CLP, common lymphoid progenitor cells; CMP, common myeloid progenitor cells; GMP, granulocyte macrophage progenitor cells; LMPP, lymphoid-primed multipotent progenitor cells; MEP, megakaryocyte erythroid progenitor cells; MPP, multipotent progenitor cells.

We next set out to assess the expression of various markers at different differentiation stages within the HSPC compartment. CLL-1 was expressed at particularly high levels in in various HSPC compartments of MDS-EB patients, including HSCs, MPPs, LMPPs, CMPs, and MEPs (Fig 2). This aberrant CLL-1 expression was also observed in the aggregate compartments of CD34+ and CD34+CD10-CD38- cells, indicating that aberrant CLL-1 expression can be detected using smaller antibody panels.

**Fig 3:**
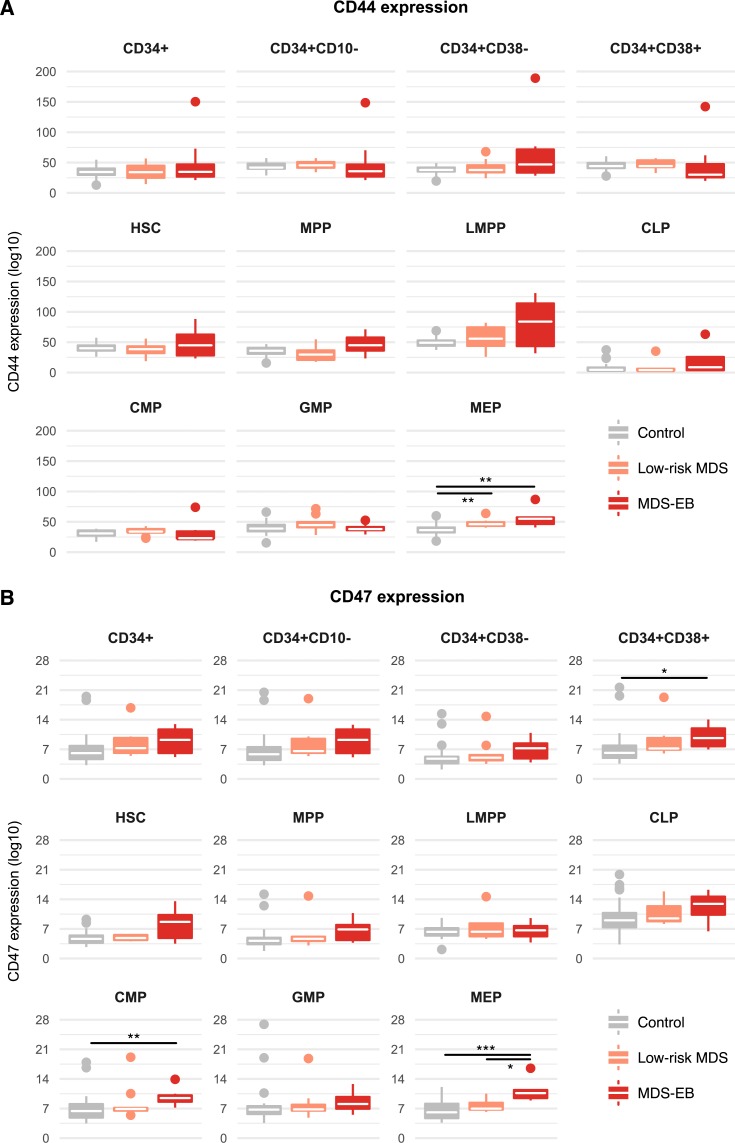
CD44 and CD47 are upregulated in specific hematopoietic compartments of patients with MDS with excess blasts. Expression of CD44 **(a)** and CD47 **(b)** was assessed in hematopoietic stem and progenitor cell compartments of control, low-risk and MDS-EB patients. White crossbars represent the median expression and boxplots hinges represent the first and third quartiles. *p<0.05, **p<0.01, ***p<0.001; CLP, common lymphoid progenitor cells; CMP, common myeloid progenitor cells; GMP, granulocyte macrophage progenitor cells; HSC, hematopoietic stem cells; LMPP, lymphoid-primed multipotent progenitor cells; MEP, megakaryocyte erythroid progenitor cells; MPP, multipotent progenitor cells.

We next analyzed the expression of CD44 and CD47 in myelodysplastic HSPCs. In both patients with low-risk MDS and patients with MDS-EB CD44 was upregulated on MEPs (Fig 3A).

The assessment of CD47 showed aberrant overexpression in CMPs and MEPs of patients with MDS-EB, which was also evident in the CD34+CD10-CD38+ parent compartment (Fig 3B). There was also upregulation of CD47 on HSC. However, this difference did not reach statistical significance (p = 0.07).

### Low-risk MDS and MDS-EB show aberrant expression of CD13/CD33

**Fig 4:**
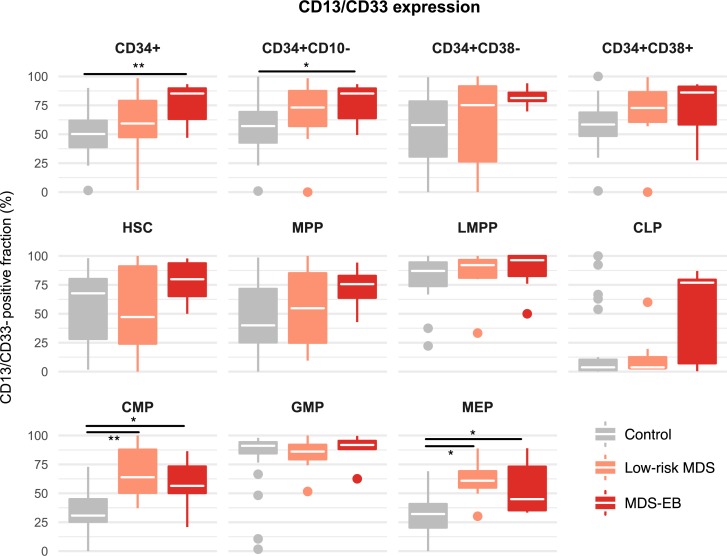
Combined expression of CD13/CD33 in MDS is upregulated in common myeloid progenitor cells (CMP) and megakaryocyte erythroid progenitor cells (MEP). The combined expression of CD13 and CD33 was assessed in hematopoietic stem and progenitor cell compartments of control versus MDS patients. White crossbars represent the median expression and boxplots hinges represent the first and third quantiles. *p<0.05, **p<0.01, ***p<0.001; CLP, common lymphoid progenitor cells; GMP, granulocyte macrophage progenitor cells; HSC, hematopoietic stem cells; LMPP, lymphoid-primed multipotent progenitor cells; MPP, multipotent progenitor cells.

We next assessed the combined expression of the myeloid markers CD13 and CD33. Aberrant upregulation of CD13/CD33 was evident in MDS-EB patients in the progenitor compartments of both CMPs and MEPs (Fig 4). Importantly, this aberrant upregulation was also evident in patients with low-risk MDS. In MDS-EB patients these changes were reflected in the CD34+ and CD34+CD10- progenitor populations as a whole. In low-risk MDS patients, no significant changes were observed in the CD34+ and CD34+CD10- populations, highlighting that more careful phenotypic dissection of these aggregate compartments enhances detection sensitivity in such patients.

### Reduced ALDH expression in the CD34+CD10-CD38- compartment in MDS with excess blasts

**Fig 5:**
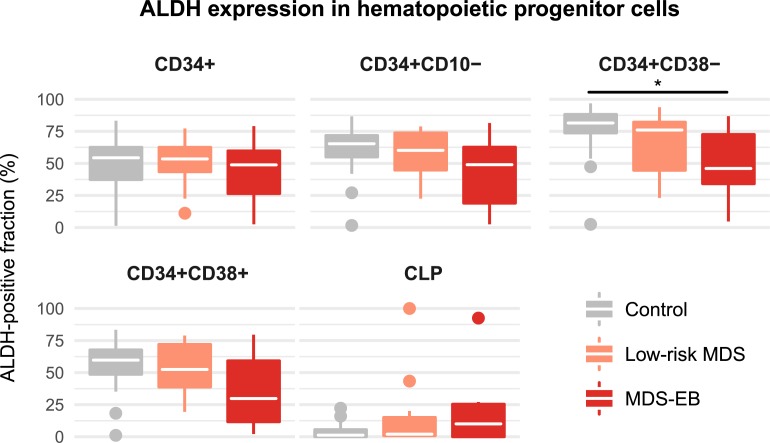
ALDH expression is diminished in CD38-negative hematopoietic progenitor cells in MDS with excess blasts. The expression of ALDH was assessed in common lymphoid progenitor cells (CLP), and compartments of CD34+, CD34+CD10-, CD34+CD38- and CD34+CD38+ cells. White crossbars represent the median expression and boxplots hinges represent the first and third quartiles. *p<0.05.

We next aimed to assess the expression of ALDH. Interestingly, in patients with MDS-EB there was a significant reduction of ALDH-expressing cells in the CD34+CD10-CD38- compartment (Fig 5).

## Discussion

Myelodysplastic syndromes have recently been established to be propagated by cancer stem cells with the capacity of disease initiation, self-renewal, and resistance to antineoplastic therapy [1–5]. A potentially curative therapeutic approach requires targeting this cancer stem cell compartment, phenotypically resembling immature, uncommitted physiologic HSC [6]. Here, we employed a modification of previously published gating strategies to assess the hematopoietic architecture as well as the expression of aberrant antigens in 20 patients with MDS. This gating strategy allowed for the detection of aberrant immunophenotypes at distinct differentiation stages within the hematopoietic progenitor cell compartment and not only in CD34+ cells as a whole.

Several studies have reported the aberrant expression of multiple antigens by cancer stem cells in AML, including CLL-1, CD47, CD44, and ALDH [11,20–22]. Targeting CLL-1, CD47, and CD44 showed promise in preclinical models of AML [21,23,24]. The expression of many of these antigens has been assessed in MDS in less specifically defined cell compartments, mostly in the CD34+CD38- compartment containing HSCs, MPPs, and LMPPs [2,22,25–27].

For CLL-1 we are able to show aberrant expression in HSCs of patients with MDS-EB. This suggests that CLL-1 has disease eliminating potential as a therapeutic target. Whether this is also the case for CD47 will have to be addressed in additional studies. In contrast, we show that increased expression of CD44 on CD34+CD38- cells in MDS-EB [19] is caused by MPPs and LMPPs but not HSC. This suggests that targeting CD44 might not be sufficient to eradicate MDS propagating clones.

Loss of ALDH expression has previously been found to be characteristic of leukemic stem cells in AML but has not been assessed in MDS so far [11,28]. Here, we show that, analogous to AML, patients with MDS-EB exhibit lower expression of ALDH in the CD34+CD38- compartment, further underpinning the biological relation between the two diseases and providing an additional marker to identify myelodysplastic stem cells. Future studies will need to establish whether MDS propagating cells are enriched in the ALDH-low compartment in analogy to AML.

Most aberrant markers are upregulated only in patients with MDS-EB, suggesting that they are not useful for the diagnosis of low-risk MDS. In this study, we observed an upregulation of the myeloid antigens CD13/CD33 on CMPs and MEPs of patients with both low-risk MDS and MDS-EB, confirming this marker combination to be useful in the diagnosis of low-risk MDS [18,29]. CD13 and CD33 have been suggested as a potential therapeutic target in MDS [17–19]. Our data, while not allowing for the distinction between CD13 and CD33, suggest that targeting either marker might not eradicate the more immature, disease propagating compartments. Surprisingly, while not statistically significant, we also observed variable degrees of CD13/CD33 expression on CLPs of MDS-EB patients. Hypothetically, this can be explained by myelodysplastic HSCs also giving rise to lymphoid progeny or by aberrant expression of CD10 on myeloid cells. More comprehensive phenotypic characterization of this compartment than in this myeloid-focused study is warranted in future studies.

Certain caveats potentially complicate antigen targeting therapy in MDS. First, eradication of the entire disease clone requires expression of the target on all cancer propagating stem cells. However, in the case of CLL-1, a recent study showed that only a subset of MDS stem cells aberrantly expresses this antigen, indicating that CLL-1 targeting might not completely eradicate the MDS propagating compartment [25]. Second, in order to minimize toxicity, the expression of the target antigen should ideally be restricted to malignant cells. This is not the case for most antigens, whose expression can also be detected on more mature hematopoietic cells (as in the case of CLL-1) or to a lower degree also on physiologic HSCs [2,22,26]. Depleting more mature physiologic hematopoietic cells while sparing physiologic HSCs as predicted for CLL-1 targeting should pose less a problem, as evident in long-established therapies such as the depletion of mature B cells in CD20+ B cell leukemia/lymphoma. In the case that the target antigen is also expressed on physiologic HSCs, differences in the expression levels might provide for a therapeutic window.

Previous studies showing that myelodysplastic hematopoiesis maintains a hierarchical organization employed stringent exclusion of lineage-positive cells [2,4,5]. However, lineage markers have been demonstrated to be aberrantly expressed in MDS stem and progenitor compartments [16–19]. Therefore, to address this concern, we here employed an alternative gating approach without exclusion of lineage-positive cells. We found that patients with MDS-EB exhibited expanded MPP and GMP compartments while the MEP compartment was reduced in both low-risk MDS and MDS-EB relative to the CD34+CD10-CD38+ compartment. Importantly, in patients with MDS-EB we also showed an expansion of the CD90-CD45RA+ LMPP compartment, which is enriched for leukemic stem cells in AML and has previously not specifically been assessed in MDS [12]. Of note, some of the antigens used to define hematopoietic progenitor compartments including CD123 can also be aberrantly expressed in MDS [17]. These findings as well as the approach regarding exclusion of lineage+ cells are important factors potentially accounting for discrepancies between studies assessing antigen expression in myelodysplastic hematopoiesis.

Certain study limitations need to be considered in the interpretation of our findings. First, the sample size was relatively small, in particular in light of the heterogeneity of MDS. As such, it is not possible to draw conclusions about phenotypic alterations in specific MDS subsets and to reliably assess the generalizability of our findings. Most phenotypic alterations described in this study were only detected in MDS-EB, with low-risk MDS often showing a trend with no statistical significance. It is conceivable that our study was underpowered to reliably detect alterations in these low-risk cases. Second, given their low abundance in the bone marrow, the number of recorded events for certain bone marrow progenitor subpopulations was low. Therefore, we cannot definitely decide whether the relatively high variability in the expression of several markers is due to the biological heterogeneity of MDS or due to a sampling bias which might have occurred in such a small population. In light of these limitations future studies are required to confirm our findings in larger patient cohorts.

**Fig 6:**
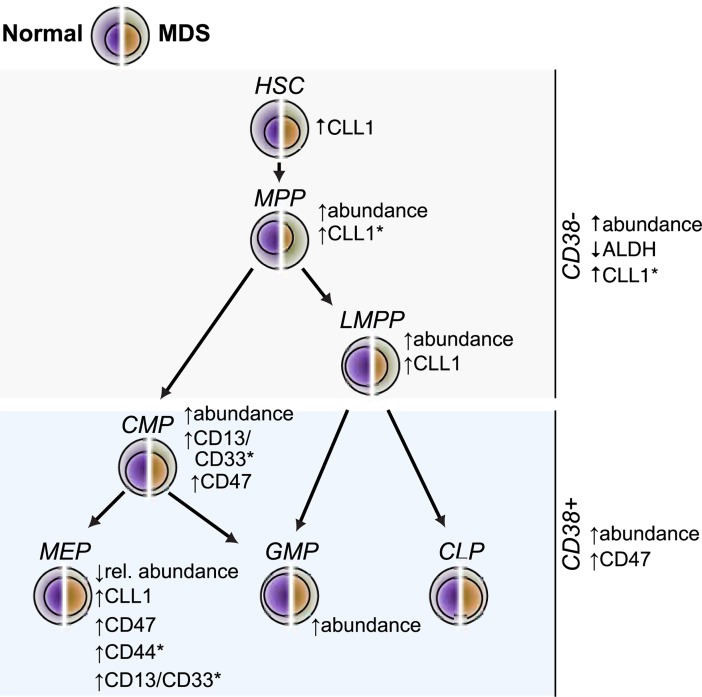
Overview of aberrantly expressed markers and proportions of myelodysplastic hematopoietic stem and progenitor cell compartments. All annotated changes refer to observations in patients with MDS with excess blasts. Alterations annotated with (*) were also observed in MDS patients without excess blasts. CLP, common lymphoid progenitor cells; CMP, common myeloid progenitor cells; GMP, granulocyte macrophage progenitor cells; HSC, hematopoietic stem cells; LMPP, lymphoid-primed multipotent progenitor cells; MEP, megakaryocyte erythroid progenitor cells; MPP, multipotent progenitor cells.

In summary, we identified particular differentiation stages of dysplastic hematopoiesis at which CLL-1, CD44, and CD47 were aberrantly expressed and at which ALDH was downregulated in MDS-EB and we showed that the myeloid markers CD13/CD33 were aberrantly expressed on CMPs and MEPs in both low-risk and MDS-EB patients (Fig 6). Aberrant expression at the most immature hematopoietic stem cell level could only be detected in the case of CLL-1, establishing CLL-1 as a promising therapeutic target.

## Supporting information

S1 Fig Proportion of hematopoietic stem and progenitor cell compartments in MDS versus control patients, expressed as percent of the parent population. White crossbars represent the median and boxplot hinges represent the first and third quartiles. *p<0.05, **p<0.01, ***p<0.001. CLP, common lymphoid progenitor cells; CMP, common myeloid progenitor cells; GMP, granulocyte macrophage progenitor cells; HSC, uncommitted hematopoietic stem cell; LMPP, lymphoid-primed multipotent progenitor cells; MEP, megakaryocytic erythroid progenitor cells; MPP, multipotent progenitor cells.
(PDF)
