# Supplementary material for: Phenotypic characterization of aberrant stem and progenitor cell populations in myelodysplastic syndromes
Source: PLoS One. 2018 May 25;13(5):e0197823. doi: 10.1371/journal.pone.0197823 (PMC5969762; doi:10.1371/journal.pone.0197823)
Supplement: S1 Fig — White crossbars represent the median and boxplot hinges represent the first and third quartiles. *p<0.05, **p<0.01, ***p<0.001. CLP, common lymphoid progenitor cells; CMP, common myeloid progenitor cells; GMP, granulocyte macrophage progenitor cells; HSC, uncommitted hematopoietic stem cell; LMPP, lymphoid-primed multipotent progenitor cells; MEP, megakaryocytic erythroid progenitor cells; MPP, multipotent progenitor cells. (PDF) [file pone.0197823.s001.pdf]

Supplementary information

Phenotypic characterization of aberrant stem and progenitor cell populations in myelodysplastic syndromes

Benjamin N. Ostendorf, Eva Flenner, Anne Flörcken, Jörg Westermann

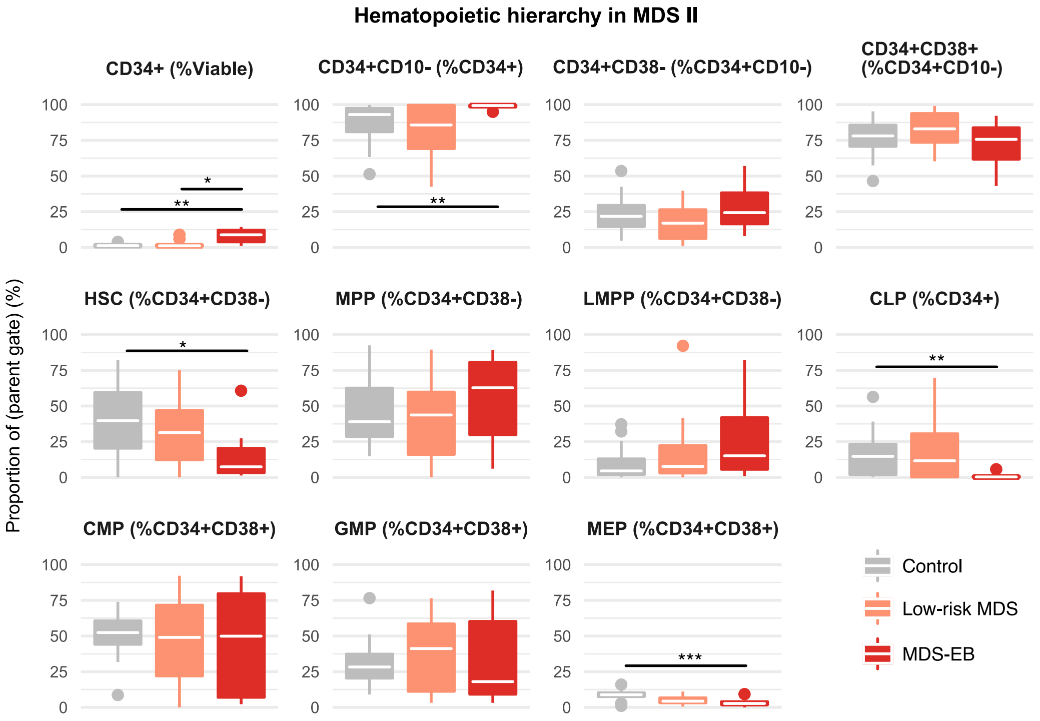

S1 Fig. Proportion of hematopoietic stem and progenitor cell compartments in MDS versus control patients, expressed as percent of the parent population. White crossbars represent the median and boxplot hinges represent the first and third quartiles. *p<0.05, **p<0.01, ***p<0.001. CLP, common lymphoid progenitor cells; CMP, common myeloid progenitor cells; GMP, granulocyte macrophage progenitor cells; HSC, uncommitted hematopoietic stem cell; LMPP, lymphoid-primed multipotent progenitor cells; MEP, megakaryocytic erythroid progenitor cells; MPP, multipotent progenitor cells.
